# Prevalence and associated factors of alexithymia among adult prisoners in China: a cross-sectional study

**DOI:** 10.1186/s12888-017-1443-7

**Published:** 2017-08-03

**Authors:** Li Chen, Linna Xu, Weimin You, Xiaoyan Zhang, Nanpeng Ling

**Affiliations:** 1 Department of Applied Psychology, Wenzhou Medical University, Wenzhou, 325035 China; 2 Department of Economics, University at Albany, State University of New York, Albany, NY USA; 3 Public Security Sub-Bureau of Huangyan, Taizhou Public Security Bureau, Huangyan, China; 4 Department of Children’s Health Care, the Second Affiliated Hospital of Wenzhou Medical University, Wenzhou, China; 5 Zhejiang Shiliping Prison, Quzhou, China

**Keywords:** Alexithymia, Duration in prison, Childhood trauma, Negative emotions, Prisoners

## Abstract

**Background:**

Prison is an extremely stressful environment and prisoners have an increasing risk of suffering from alexithymia*.* Therefore, this study aims to investigate the prevalence and associated factors of alexithymia among prisoners in China.

**Methods:**

A cross-sectional study was conducted in five main jails of the district of Zhejiang province in China, and a total of 1705 adult prisoners ultimately took part in the study. Toronto Alexithymia Scale, Childhood Trauma Questionnaire, Beck Depression Inventory, Beck Anxiety Inventory, Beck Hopelessness Scale and several short demographic questions were applied.

**Results:**

Over 30% of prisoners were classified as alexithymics and as high as 96.2% of prisoners suffered from at least one traumatic experience in their childhood, meanwhile, 81.5%, 53.4% and 85.8% were found to be positive for depression, anxiety and hopelessness symptoms respectively. Education, childhood trauma, negative emotional symptoms including depression, anxiety and hopelessness of the respondents, were negatively or positively associated with alexithymia among prisoners.

**Conclusions:**

The results indicated that high prevalence of alexithymia among prisoners is linked with their level of education, experience of childhood trauma and symptoms of negative emotions. Accordingly, the findings in our study can be used for prevention and intervention of alexithymia among prisoners.

**Electronic supplementary material:**

The online version of this article (doi:10.1186/s12888-017-1443-7) contains supplementary material, which is available to authorized users.

## Background

Alexithymia is a personality construct characterized by the subclinical inability to identify and describe emotions in the self [1, 2]. The core characteristics of alexithymia are as follows: (1) difficulty identifying feelings and distinguishing between feelings and the bodily sensations of emotional arousal, (2) difficulty describing feelings to other people, (3) constricted imaginal processes, as evidenced by a scarcity of fantasies, (4) a stimulus-bound, externally oriented cognitive style [3]. This four-factor characterization of alexithymia has become the standard for describing the construct [4]. Thus, individuals suffering from alexithymia mostly have difficulty in emotional self-regulation. Meanwhile, a growing body of evidence has proved that lower level of emotion regulation is strongly related to both low level of social competence and the expression of socially appropriate emotions, which also impaired individual’s psychological mindedness and emotional intelligence [1, 2, 5–7]. Furthermore, Alexithymia implies problems in the capacity for mentalization by impairing the ability to understand that one has one’s own thoughts and feelings, separate from others’ thoughts and feelings, and that those mental processes motivate behavior in oneself and in others [4]. Therefore, alexithymics have difficulty in emotional self-regulation and mentalizing.

Published studies indicated that people with stressful experience or in stressful situation have higher level of alexithymia, especially patients... For example, alexithymia frequently co-occurs with stress-related illness including severe physical illnesses, such as coronary heart disease [8–10], hypertension [11, 12], diabetes [13–17] and psoriasis [18–20], or some psychiatric illnesses, such as eating disorders [21–24], autism spectrum disorders [25–28], panic disorder [29–34] and post-traumatic stress disorder [35–37]. These studies not only have significantly advanced current knowledge concerning the prevalence of alexithymia among different patients who are suffering from stress, but also have motivated new important research questions. For instance, imprisonment is also an extremely stressful experience. In prison, prisoners are forcibly confined in a limited space, and consequently, their freedom and interpersonal relationships are acutely restricted [38–41]. Is it possible for prisoners to have higher incidence and level of alexithymia compared with the general population? Meanwhile, relevant studies have repeatedly demonstrated higher rates of mental problems among prisoners than the general population, such as depression, anxiety, and childhood trauma [42, 43]. Could these mental problems be strong risk factors for alexithymia among prisoners? Based on the above analysis, our study seeks to go beyond existing studies to further examine the prevalence and severity of alexithymia among prisoners by making the following two specific efforts.

First, we focus on the prevalence of alexithymia among adult prisoners in Chinese context. In the past three decades, the prevalence of alexithymia in China has received much attentions in different groups. The first empirical and wide-scale study on alexithymia in China was carried out among college students in 1991. It was found that 11.88% of 488 investigated college students suffered from alexithymia [44]. The following investigations and research have focused largely on patient group with different medical or psychiatric illness [45–48]. However, studies on alexithymia among prisoners are limited to male prisoners or small-scale participants and do not reveal the overall prevalence of alexithymia among prisoners, especially in the context of Chinese society [49, 50]. In china, over the past 35 years, China’s crime rate has increased dramatically along with the noteworthy increase in economic growth [51–53]. Provincial crime rate in China has increased from 4.76 per 10,000 persons in 1997 to 7.42 in 2007 [51]. Furthermore, according to the International Center for Prison Studies at King’s College in London, China has the second-largest number of prisoners (1.51 million, for a rate of 117 per 100,000) in the world. Therefore, it is necessary to investigate the prevalence of alexithymia among prisoners.

Secondly, we would explore the risk and protective factors for alexithymia among prisoners. On the one hand, we evaluate the relationship between some sociodemographic factors and alexithymia among prisoners, especially the experience of imprisonment. For example, is there any significant association between the experience of imprisonment and alexithymia among prisoners? According to previous studies, the positive linear relationship between illness experience and alexithymia has been well studied among patients with chronic diseases [54, 55]. D. De Berardis’group, for instance, evaluated relationship between alexithymia and duration of illness in a sample of adult outpatients with obsessive-compulsive disorder. They found that alexithymics showed a longer duration of illness and were more likely to have a chronic disease than nonalexithymics [55]. Later, Celikel and Saatcioglu surveyed 30 female chronic pain patients and 37 healthy females and the results showed that chronic pain patients were significantly more alexithymic than the controls, and there was a positive correlation between the level of alexithymia and the duration of pain [54]. Similar to the experience of illness, we hypothesized that the experience of imprisonment could have positive association with the severity of alexithymia among prisoners.

On the other hand, we explore the associated factors for alexithymia from two personal experiences of prisoners, childhood trauma and negative emotions. Childhood trauma refers to the experience of an event by a child that is emotionally painful or distressful, which often results in lasting mental and physical effects. A great deal of evidence has demonstrated that childhood trauma is associated with a wide variety of undesirable outcomes, and one possible effect of childhood trauma is alexithymia [43, 56, 57]. Specifically, childhood trauma can impair the affect-regulating capacity, which may subsequently lead to alexithymia [56]. Because affect-regulating capacity is learned in interaction with caregivers in the childhood, chronic interpersonal trauma in early developmental stages could disrupt the development of adaptive emotion regulation [58]. For example, in two studies, Shipman and colleagues, found that compared to nonmaltreated children, maltreated children were less able to understand and regulate emotions, they also received less emotional support and have more peer rejection [59, 60]. Furthermore, those experienced childhood trauma lack the proper vocabulary to articulate and make sense of the experiences, including the powerful and sometimes overwhelming feelings involved. These studies suggest that maltreated children show deficits or delays in understanding and regulating emotions. Another personal experience is negative emotion. Negative emotion is defined as an unpleasant or unhappy emotion that is evoked to express a negative affect towards an event or a person, usually including depression, anxiety, and hopelessness. Previous studies have found that alexithymia is associated with various negative emotions of clinical patient groups [24, 54, 56]. Clinical patients, especially patients with chronic diseases, are suffering from severe and prolonged stress due to illnesses, which cause the patients to a high level of negative emotions, especially anxiety and depression. Meanwhile, based on the secondary alexithymia theory, alexithymia can be viewed as a defense or strategy to cope with emotional pain, which means people with negative emotions, would be possible with high level of alexithymia [61, 62]. Despite the increased awareness of the association between alexithymia and childhood trauma or negative emotions in clinical samples, relatively few studies have been undertaken for prisoners. Prisoners, being kept under involuntary restraint prisons for a long time, are with much higher level and prevalence of childhood trauma and negative emotions. Therefore, we might hypothesize that childhood trauma and negative emotions are risk factors for alexithymia among prisoners.

The aims of the present paper were to (1) investigate the prevalence of alexithymia among prisoners in China and (2) identify the risk and protective factors for alexithymia among prisoners.

## Methods

### Participants

A total of 1705 adult prisoners detained in five main jails of the district of Zhejiang in China took part in the study. The final eligible participants were identified by the following steps.

Firstly, we used a multi-stage probability sampling method to obtain a representative sample. We used sub-prison and cellblock as stratum, and then selected cells by cluster sampling in each stratum (in China, the organization framework of each prison could be divided into four levels. The first level is the prison; the second level is the sub-prison and one prison could be consisted of several sub-prisons; the third level is the cellblock and one sub-prison could be consisted of several cellblocks; the fourth level is cells with 8–12 prisoners and one cellblock could be consisted of several cells). In total, 1942 prisoners were selected from 175 cells.

Secondly, four criteria have been used to select eligible participants from the 1942 prisoners: (1) prisoners had to be aged ≥18 years; (2) prisoners could speak or read Chinese characters; (3) we included sentenced prisoners but excluded remand prisoners; (4) prisoner was willing to participate in the study and to sign the informed consent.

Hence, 1864 adult prisoners were eligible for the study and consent with the study procedures, and then1705 made valid replies, yielding a response rate of 91.46%. Details of socio-demographic characteristics of the sample are displayed in Table 1. Of a total 1705 prisoners, 1059 (62.1%) were male and 646 (37.9%) were female. Over two-third of them (1210, 71%) were under the age of 35 years old and lived in rural communities (1167, 68.4%). The prisoners were more likely to be under-educated with only 96 (5.6%) of them had education level of college or above. Over half of them were single (957, 56.1%). The information of maximum sentence length and duration in prison in our sample is listed in Table 1 (A﻿dditional file 1: Data used in this paper).

**Table 1:** Socio-demographic characteristics of the sample (*N* = 1705)

Variables		Number	Percent
Gender	Male	1059	62.1
	Female	646	37.9
Age	18–24	625	36.7
	25–34	585	34.3
	35–44	298	17.5
	45–54	96	5.6
	>54	23	1.3
	Missing	78	4.6
Education level	Primary school or below	455	26.7
	Secondary school/technical school	1140	66.9
	College or above	96	5.6
	Missing	14	0.8
Location of residence	Urban	504	29.5
	Rural	1167	68.4
	Missing	34	2.1
Marital status	Single	957	56.1
	Married/Cohabiting	549	32.2
	Divorced	172	10.1
	Windowed	14	0.8
	Missing	13	0.8
Maximum sentence length (months)	36 or less	426	25.0
	37–60	408	23.9
	61–120	419	24.6
	>120	291	17.1
	suspended death/life imprisonment	126	7.4
	Missing	35	2.1
Duration in prison (months)	12 or less	374	21.9
	13–24	601	35.2
	25–48	345	20.2
	49–120	253	14.8
	>120	83	4.9
	Missing	49	2.9

### Procedure

A cross-sectional survey was conducted between May and December, 2014 among adult prisoners in five prisons (Zhejiang Jinhua Prison, Zhejiang No.3 Prison, Zhejiang Shilifeng Prison, Zhejiang Women Prison, and Zhejiang Shiliping Prison) in three cities: Quzhou, Jinghua and Hangzhou, Zhejiang Province, China. We chose these three cities as our study sites since Zhejiang was one of the areas with largest number of prisoners in China, i.e. over 0.1 million prisoners; and more than two-thirds of prisoners in this area are currently held in prisons located in Quzhou, Jinghua and Hangzhou.

The present study consisted of the following steps. First, in the pilot study, the pre-test was conducted with a convenience sample of 32 adult prisoners from the target population to evaluate clarity, comprehensiveness, and acceptability of questionnaires. Some amendments were made prior to the initial delivering. Secondly, in the formal study, after fully understanding the purpose and the procedure of the study, the eligible participants filled out the 20-min questionnaires in quiet and comfortable reading rooms in prisons (one to six participants at one time). The questionnaires were administered by trained researchers, including prison counselors and faculty members from Wenzhou Medical University, who had been provided systematic training before formal study. The questionnaires were anonymous and all participants took part in the study voluntarily. The study was reviewed and approved by the Ethics Committee of Wenzhou Medical University.

### Measures

#### Socio-demographics

A demographic questionnaire elicited basic background information, including age, gender, marital status, education, location of residence (rural vs. urban), crime type, maximum sentence length (months), duration in prison (months).

#### Alexithymia

Alexithymia was measured by the Chinese version [63] of 20-item Toronto Alexithymia Scale (TAS-20) [1]. The TAS-20 is a self-reported scale comprised of 20 items to assess three factors of alexithymia: (1) Difficulties Identifying Feelings (DIF) (e.g., I am often confused about what emotion I am feeling); (2) Difficulties Describing Feelings (DDF) (e.g., It is difficult for me to find the right words for my feeling); (3) Externally Oriented Thinking (EOT) (e.g., Looking for hidden meaning in movies or plays distracts from their enjoyment). Each of these 20 items is rated on a five-point Liker scale (1 = “strongly disagree” to 5 = “strongly agree”). According to the classification criteria reported by Gulec et al., scores of 61 or above indicate alexithymia, scores of 60 and below indicate no alexithymia [64]. Both exploratory and confirmatory factor analyses supported the construct validity of the three subscales.,In addition, The Cronbach’s alphas for the subscales range from 0.67–0.88, the Cronbach’s alphas for the whole scale was 0.86 and a test-retest reliability coefficient of the TAS was 0.81 [63]. This scale has been found to discriminate effectively between high-alexithymia and low-alexithymia individuals. In this study, A Cronbach’s alpha of all items was 0.83 and shows high level of internal consistency, suggesting that items are homogeneous.

#### Childhood trauma

Childhood trauma was evaluated by the 28-item Chinese version of Childhood Trauma Questionnaire (CTQ) [65]. The CTQ is a 28-item self-reported instrument and respondents are asked to rate the severity of different types of childhood traumas using a 5-point scale ranging from 1 (never) to 5 (very often). Items are combined to form five subscales: emotional abuse, physical abuse, sexual abuse, emotional neglect, and physical neglect. The total CTQ score takes into account the severity of multiple forms of abuse and neglect. Previous research demonstrates the Chinese version of CTQ has strong internal consistency (coefficient alpha = 0.77) and a two-month test-retest reliability of the overall CTQ (*r =* 0.75). In addition, the internal consistency of each subscale was adequate (alpha coefficients were from 0.47 to 0.68) [66]. The CTQ has proven to be a valid measure of childhood trauma with adults and adolescents Likewise, the scale performed well with this study’s adults (Coefficient Alpha = 0.89).

#### Symptoms of negative emotion

##### Depression

Depression symptom severity was measured by 21-item Beck Depression Inventory-Second Edition (BDI-II) [67]. Each of the 21 questions on the BDI-II is rated on a four-point scale ranging from 0 to 3. Each question is scored: 0 = symptom absent; 1 = symptom present; 2 = moderate symptom; and 3 = severe symptom. The total score can range from 0 to 63. Higher scores indicate a higher degree of depressive symptoms. Previous research demonstrates the BDI-II has good internal consistency (Coefficient Alpha = 0.91) and good test–retest reliability (*r* = 0.93). The BDI-II also has good concurrent validity with the Hamilton Rating Scale for Depression (*r* = 0.71) [68]. In the current sample, BDI-II scores show moderate internal consistency (Cronbach’s alpha = 0.81).

##### Anxiety

Severity of anxiety symptom was assessed using Beck Anxiety Inventory (BAI) [69], which includes 21 items graded from 0 (not at all) to 3(severely). BAI scores were categorized into normal (0–9), mild anxiety (10–16), moderate anxiety (17–29) and severe anxiety (30–63). The total score can range from 0 to 63. Higher scores indicate more severe anxiety. It has a high internal consistency (Cronbach’s alpha = 0.92) and test-retest reliability of 0.75.

This scale has been found to discriminate effectively between high-anxiety and low-anxiety individuals. In this study, the alpha for the scale is 0.88.

##### Hopelessness

Severity of hopelessness symptom was assessed using Beck Hopelessness Scale (BHS) [70]. It consists of 20 true-or-false items assessing three factors: feelings about the future, loss of motivation, and future expectations. Total score of BHS is a sum of item responses and can range from 0 to 20, Higher scores indicate a higher degree of pessimism and hopelessness. BHS scores were categorized into normal (0–3), mild hopelessness (4–8), moderate hopelessness (9–14) and severe hopelessness (15–20). The total score can range from 0 to 20. Cronbach’s alpha of the Chinese version of BHS was 0.85. In this study, the alpha for the scale is 0.93.

### Data analysis

First of all, socio-demographic characteristics of the sample were described by the number and the percentage of each category for categorical variables. Secondly, we calculated prevalence estimates of alexithymia, childhood traumas and symptoms of negative emotions among the adult prisoners. Thirdly, independent sample t-tests were conducted for total scale and subscale scores of TAS-20 and CTQ between our adult prisoners sample and different Chinese adult norms. Fourthly, Pearson’s correlation analysis was used to check the correlations between variables. Finally, multivariate binary logistic regression was used to determine the risk factors for alexithymia of prisoners, and crude odds ratios and adjusted odds ratios (OR) and 95% confidence intervals (CIs) for OR were calculated. In logistic regression modeling, the dependent variable was whether the prisoner was alexithymic or not ((if yes, then y = 1; otherwise, y = 0). The demographic information (age, gender, education, marital status and region of origin), imprisonment information (maximum sentence length and duration in prison), symptoms of negative emotion information (depression, anxiety and hopelessness) and experience of childhood trauma of prisoners were considered as the independent variables. All statistical analyses were performed with the use of SPSS statistics package (version 18.0) and all reported *P*-values are 2-tailed with statistical significance set at 0.05.

## Results

### Prevalence of alexithymia among the prisoners

In general, out of 1705 adult prisoners, over one thirds of the respondents (31.4%, 95% CI:30.3–32.5%) were classified as alexithymics (*M* = 66.17, *SD* = 4.49). In order to determine whether prisoners are at a higher risk of more severe alexithymia compared with the adult norm in China, independent t-tests were conducted on TAS-20 total score and subscales scores. As shown in Table 2, scores of three subscales and total score of TAS-20 were significantly higher than the adult norms of 2003 and 2007 in China.

**Table 2:** Comparison of TAS-20 total score and subscales scores between the adult prisoners and the adult norm of 2003 and 2007

		DIF	DDF	EOT	TAS-20 TOTAL
Samples of adult prisoners	(*N* = 1705)	19.95 ± 5.03	14.59 ± 2.87	21.93 ± 3.34	56.47 ± 8.61
The adult norm of 2003^a^	(*N* = 416)	17.33 ± 4.70	13.44 ± 3.29	18.91 ± 4.01	49.68 ± 9.24
*t* ^c^		21.52^***^	16.56^***^	37.36^***^	32.60^***^
The adult norm of 2007^b^	(*N* = 870)	16.26 ± 4.75	13.26 ± 3.51	19.12 ± 3.86	48.65 ± 9.24
*t* ^d^	30.31^***^	19.15^***^	34.77^***^	37.54^***^	

^a^For the data source, see reference [61] (*The Chinese version of the TAS-20: reliability and validity*)

^b^For the data source, see reference [55] (*Cross-cultural validation of a Chinese translation of the 20-item Toronto Alexithymia Scal*e)

^c^Comparison of TAS −20 total score and subscales scores between the adult prisoners and the adult norm of 2003

^d^Comparison of TAS −20 total score and subscales scores between the adult prisoners and the adult norm of 2007

*p < 0.05 **p < 0.01 ***p < 0.001

### Prevalence of childhood trauma among the prisoners

In this study, 1640 (96.2%, 95% CI 95.7–96.67%) of the respondents reported suffering from at least one type of childhood trauma. Specifically, The most frequently reported childhood trauma by adult prisoners was emotional neglect (86.8%, 95% CI 85.9–86.8%), followed by physical neglect (82.4%, 95% CI 81.4–83.3%), emotional abuse (70.4%, 95% CI 69.3–71.5%), physical abuse (47.2%, 95% CI 0.46–48.4%) and sexual abuse (43.6%, 95% CI 42.4–44.8%).

To determine whether prisoners would report elevated levels of childhood trauma compared with the adult norm in China, independent t-tests were conducted with each CTQ total score and subscales scores. As shown in Table 3, scores of five subscales and total score of CTQ were significantly higher than the adult norm of 2005 in China.

**Table 3:** Comparison of CTQ total score and subscales scores between the adult prisoners and college students of 2005

		Emotional abuse	Physical abuse	Sexual abuse	Emotional neglect	Physical neglect	CTQ TOTAL
Samples of adult prisoners	(*N* = 1705)	7.99 ± 3.32	7.28 ± 3.41	6.80 ± 3.13	11.84 ± 5.23	9.41 ± 3.78	43.33 ± 14.38
The adult norm of 2005^a^	(*N* = 593)	6.54 ± 2.11	5.69 ± 1.73	5.58 ± 1.37	9.65 ± 4.06	8.43 ± 2.60	35.89 ± 8.25
*t* ^b^		18.02^***^	19.27^***^	16.09^***^	17.29^***^	10.78^***^	21.34^***^

^*^ *p* < 0.05 ^**^ *p* < 0.01 ^***^ *p* < 0.001

^b^Comparison of CTQ total score and subscales scores between the adult prisoners and the adult norm of 2005

^a^For the data source, see reference [91] (*Initial reliability and validity of childhood trauma questionnaire applied in Chinese college students*)

### Prevalence of symptoms of negative emotion among the prisoners

According to the Beck Inventories, the mean scores for depression, anxiety and hopelessness were 19.41 ± 10.75, 12.05 ± 10.86 and 7.64 ± 4.03 respectively.

According to their depression level, the overall prevalence of depression was found to be 81.5%. Based on BDI questionnaire cut-off scores, 316 adult prisoners (18.5%) scored as normal (0–13), 360 (21.1%) as mild (14–19), 684 (40.1%) as moderate (20–28), and 345 (20.2%) as severe (29+) depression. Among those with depression, nearly a half (40.1%) had moderate degree of depression. According to their anxiety level, the overall prevalence of anxiety was found to be 53.4%. Based on BAI questionnaire cut-off scores, it was found that 469 (27.5%), 267 (15.7%) and 133 (7.8%) suffer from mild, moderate and severe anxiety states respectively. According to their hopelessness level, the overall prevalence of hopelessness was found to be 85.8%. Based on BHS questionnaire cut-off scores, it was found that 610 (35.8%), 518 (30.4%) and 82 (4.8%) suffer from mild, moderate and severe hopelessness states respectively. Three symptoms of negative emotions appear to show similar characteristics on the frequency distributions, in which participants mostly had mild or moderate degree of symptoms. Details of prevalence of depression, anxiety and hopelessness among the samples are displayed in Table 4.

**Table 4:** Prevalence of depression, anxiety and hopelessness among the adult prisoners

levels of symptom	depression		anxiety		hopelessness	
	Numbers	%	Numbers	%	Numbers	%
Symptom absent	316	18.5	795	46.6	242	14.2
Mild symptom	360	21.1	469	27.5	610	35.8
Moderate symptom	684	40.1	267	15.7	518	30.4
Severe symptom	345	20.2	133	7.8	82	4.8

### Factors associated with alexithymia

Pearson correlation matrix, as shown in Table 5, TAS-20 total score has a significant and positive correlation with duration in prison (*r* = 0.15, *p* < 0.01), CTQ total score (*r* = 0.21, *p* < 0.01) and five subscales scores (Emotional abuse, *r* = 0.20, *p* < 0.01; Physical abuse, *r* = 0.15, *p* < 0.01; Sexual abuse, *r* = 0.11, *p* < 0.01). Emotional neglect, *r* = 0.13, *p* < 0.01; Physical neglect, *r* = 0.20, *p* < 0.01), and negative emotions (for BDI, *r* = 0.29, *p* < 0.01; for BAD, *r* = 0.25, *p* < 0.01; for BHS, *r* = 0.30, *p* < 0.01). Meanwhile, TAS-20 total score has a significant and positive correlation with education of prisoners (*r* = 0.18, *p* < 0.01).

**Table 5:** Intercorrelations among variables used in this study (*N* = 1705)

	Variables	1	2	3	4	5	6	7	8	9	10	11	12	13	14	15	16	17	18	19	20
1	gender	1																			
2	age	−.01	1																		
3	education	.22**	.02	1																	
4	Location of residence	−.20*	−.22*	−.21*	1																
5	Marital status	.16**	.53**	.07**	−.16**	1															
6	Maximum sentence length	−.19**	.18**	−.03	−.03	−.02	1														
7	Duration in prison	−.27**	.24**	−.09**	.01	−.07**	.76**	1													
8	DIF	.03	.02	−.12**	.03	.04	.07**	.07**	1												
9	DDF	−.01	.03	−.15**	.05*	.04	.03	.05	.62**	1											
10	EOT	−.09**	−.04	−.16**	.05*	−.07**	.04	.12**	.22**	.21**	1										
11	TAS total score	−.02	−.01	−.18**	.04	.01	.07**	.15**	.87**	.78**	.58**	1									
12	BDI	−.03	.05	−.06*	.03	.02	.19**	.16**	.29**	.21**	.10**	.29**	1								
13	BAI	−.03	.01	−.11**	.06*	−.00	.14**	.14**	.26**	.21**	.07**	.25**	.50**	1							
14	BHS	−.11**	.01	−.15**	.07**	−.03	.15**	.10**	.23**	.21**	.20**	.30**	.49**	.30**	1						
15	Emotional abuse	−.13**	−.06*	−.17**	.06*	−.08**	.08**	.11**	.18**	.15**	.11**	.20**	.20**	.27**	.22**	1					
16	Physical abuse	−.21**	−.05*	−.18**	.09**	−.08**	.13**	.16**	.10**	.12**	.14**	.15**	.19**	.21**	.20**	.68**	1				
17	Sexual abuse	−.24**	−.01	−.19**	.08**	−.05*	.08**	.11**	.05*	.08**	.13**	.11**	.12**	.20**	.14**	.55**	.56**	1			
18	Emotional neglect	−.21**	−.10**	−.25**	.08**	−.13**	.06**	.10**	.06**	.09**	.16**	.13**	.09**	.12**	.22**	.42**	.40**	.24**	1		
19	Physical neglect	−.22**	.02	−.27**	.08**	−.04	.07**	.12**	.15**	.15**	.17**	.20**	.16**	.18**	.21**	.49**	.46**	.33**	.62**	1	*
20	CTQ total score	−.26**	−.06*	−.28**	.10**	−.11**	.11**	.14**	.14**	.16**	.19**	.21**	.19**	.25**	.26**	79**	.78**	.65**	.77**	.78**	1

* *p* < 0.05 ** *p* < 0.01 ***p < 0.001

In the regression model of alexithymia, as shown in Table 6, the associations between alexithymia and the socio-demographic characteristics, symptoms of negative emotion and childhood trauma of adult prisoners were explored further. The protective effects of education for adult prisoners were found to be significant in the experiences of alexithymia. Specifically, compared to those adult prisoners with primary school education level or lower, adult prisoners with secondary school/technical school (AOR 0.73, 95% CI:0.54–0.98) and college (AOR 0.19, 95% CI: 0.09–0.42) level were less likely to experience alexithymia. Meanwhile, adult prisoners in the group with duration in prison longer than 120 months (COR 2.16, 95% CI:1.33–3.52) is more likely to have experienced alexithymia than that in other groups. However, this association was not significant after controlling for other factors (AOR 1.44, 95% CI:0.63–3.31).

**Table 6:** crude and adjusted odds ratios (OR) and 95% confidence intervals (95% CI) of alexithymia by sociodemographic factors, symptoms of negative emotion and childhood trauma

	Alexithymia		cOR(95% CI)	*p*	aOR (95% CI)	*p*
	No (*n* = 1170) *n* (%)	Yes(*n* = 535) *n* (%)				
Gender						
Male	729 (62.3)	330 (61.7)	1.00 (reference)		1.00 (reference)	
Female	441 (37.7)	205 (38.3)	1.03 (0.83–1.27)	0.81	1.28 (0.96–1.71)	0.09
Age group						
18–24	399 (35.6)	178 (35.2)	1.00 (reference)		1.00 (reference)	
25–34	474 (42.3)	187 (37.0)	0.88 (0.69–1.13)	0.32	0.86 (0.62–1.20)	0.38
35–44	182 (16.2)	96 (19.0)	1.18 (0.87–1.60)	0.28	1.13 (0.72–1.77)	0.61
45–54	48 (4.3)	32 (6.3)	1.49 (0.92–2.42)	0.10	2.02 (1.02–3.97)	0.04
> 54	18 (1.6)	13 (2.6)	1.62 (0.77–3.38)	0.20	1.39 (0.50–3.85)	0.53
Education group						
Primary school or lower	277 (23.8)	178 (33.5)	1.00 (reference)		1.00 (reference)	
Secondary school/technical school	797 (68.5)	343 (64.5)	0.67 (0.53–0.84)	0.00	0.73 (0.54–0.98)	0.04
College or above	89 (7.7)	11 (2.1)	0.19 (0.10–0.37)	0.00	0.19 (0.09–0.42)	0.00
Location of residence						
Urban	350 (30.2)	154 (29.4)	1.00 (reference)		1.00 (reference)	
Rural	810 (69.8)	369 (70.6)	1.04 (0.83–1.29)	0.76	0.92 (0.68–1.23)	0.56
Marital status						
Single	663 (57.2)	294 (55.3)	1.00 (reference)		1.00 (reference)	
Married/Cohabiting	377 (32.5)	172 (32.3)	1.03 (0.82–1.29)	0.81	1.12 (0.79–1.56)	0.53
Divorced/Windowed	120 (10.3)	66 (12.4)	1.24 (0.89–1.73)	0.20	1.11 (0.69–1.79)	0.67
Maximum sentence length (months)						
36 or less	302 (26.3)	124 (23.7)	1.00 (reference)		1.00 (reference)	
37–60	284 (24.8)	124 (23.7)	1.06 (0.79–1.43)	0.69	1.16 (0.81–1.66)	0.43
61–120	297 (25.9)	122 (23.3)	1.00 (0.74–1.35)	0.99	0.97 (0.66–1.43)	0.88
> 120	189 (16.5)	102 (19.5)	1.31 (0.96–1.81)	0.09	1.12 (0.69–1.79)	0.65
suspended death /life imprisonment	75 (6.5)	51 (9.8)	1.66 (1.09–2.50)	0.02	1.13 (0.54–2.36)	0.75
Duration in prison (months)						
12 or less	269 (23.7)	105 (20.2)	1.00 (reference)		1.00 (reference)	
13–24	418 (36.8)	183 (35.2)	1.12 (0.84–1.49)	0.43	1.15 (0.82–1.60)	0.43
25–48	233 (20.5)	112 (21.5)	1.23 (0.89–1.69)	0.20	1.13 (0.74–1.74)	0.56
49–120	171 (15.1)	82 (15.8)	1.23 (0.87–1.74)	0.24	0.96 (0.55–1.66)	0.88
> 120	45 (4.0)	38 (7.3)	2.16 (1.33–3.52)	0.00	1.44 (0.63–3.31)	0.39
BAI						
no anxiety	599 (52.6)	196 (37.3)	1.00 (reference)		1.00 (reference)	
mild anxiety	341 (29.9)	128 (24.4)	1.15 (0.89–1.49)	0.30	0.72 (0.51–1.01)	0.05
moderate anxiety	140 (12.3)	127 (24.2)	2.77 (2.08–3.70)	0.00	1.43 (1.02–2.09)	0.04
severe anxiety	59 (5.2)	74 (14.1)	3.83 (2.63–5.59)	0.00	1.71 (1.06–2.73)	0.03
BDI						
no depression	258 (22.1)	58 (10.8)	1.00 (reference)		1.00 (reference)	
mild depression	270 (23.1)	90 (16.8)	1.48 (1.02–2.15)	0.04	1.33 (0.87–2.03)	0.18
moderate depression	470 (40.2)	214 (40.0)	2.03 (1.46–2.81)	0.00	1.62 (1.08–2.41)	0.02
severe depression	172 (14.7)	173 (32.3)	4.47 (3.14–6.38)	0.00	2.93 (1.84–4.67)	0.00
BHS						
no hopelessness	201 (20.0)	41 (9.1)	1.00 (reference)		1.00 (reference)	
mild hopelessness	443 (44.2)	167 (37.2)	1.85 (1.26–2.70)	0.00	1.56 (1.01–2.39)	0.04
moderate hopelessness	320 (31.9)	198 (44.1)	3.03 (2.08–4.44)	0.00	1.86 (1.18–2.92)	0.03
severe hopelessness	39(3.9)	43(9.6)	5.41 (3.12–9.35)	0.00	2.35(1.21–4.55)	0.01
CTQ						
Never (none)	54 (4.6)	10 (1.9)	1.00 (reference)		1.00 (reference)	
Rarely (low)	855 (73.1)	331 (61.9)	2.09 (1.05–4.15)	0.03	2.40 (1.02–5.66)	0.05
Sometimes (moderate)	238 (20.3)	166 (31.0)	3.76 (1.86–7.61)	0.00	3.63 (1.49–8.84)	0.00
Often/very often(severe)	23(2.0)	28(5.2)	6.57(2.75–15.72)	0.00	4.08(1.35–12.32)	0.01

On the other hand, symptoms of negative emotion including anxiety, depression and hopelessness were identified as significant risk factors for alexithymia. Specifically, respondents with mild anxiety (AOR 0.72, 95% CI: 0.51–1.01), moderate anxiety (AOR 1.43, 95% CI: 1.02–2.09) and severe anxiety (AOR 1.71, 95% CI: 1.06–2.73) were more likely to report alexithymia than those with minimal anxiety. Similarly, respondents with mild depression (AOR 1.33, 95% CI: 0.87–2.03), moderate depression (AOR 1.62, 95% CI: 1.08–2.41 and severe depression (AOR 2.93, 95% CI: (1.84–4.67) were more likely to report alexithymia than those with minimal depression. Also, with the increase of level of hopelessness, the odds of exposure to alexithymia increased. Compared with respondents with minimal hopelessness, those with mild hopelessness (AOR 1.56, 95% CI: 1.01–2.39), moderate hopelessness (AOR 1.86, 95% CI: 1.18–2.92 and severe hopelessness (AOR 2.35, 95% CI: 1.21–4.55) were less likely to report alexithymia.

In the same way, the experience of childhood trauma were also identified as a significant risk factor associated with alexithymia among adult prisoners. Compared to respondents without the experience of childhood trauma, those who experience childhood trauma from rarely to frequently were about over two times (AOR 2.40, 95% CI 1.02–5.66), nearly four times (AOR 3.63, 95% CI 1.49–8.84) and over four times (AOR 4.08, 95% CI 1.35–12.32) more likely to report alexithymia.

## Discussion

This study estimated the prevalence and frequency of alexithymia, childhood trauma and symptoms of negative emotion in a large sample of Chinese adult prisoners, and identified the risk and protective factors for alexithymia.

To our best knowledge, this study is the first to estimate the prevalence of alexithymia in a large sample of typical Chinese adult prisoners. Prevalence of alexithymia was found to be 31.4% among prisoners in our study. The incidence rate of alexithymia in this study is much higher than the findings of other studies, in which the alexithymia occurs in approximately 8–19% in general population [71]. Meanwhile, the prisoner sample had significantly higher alexithymia scores than Chinese adult norm of 2003 and 2007.

Both prison environmental factors and prisoner’s individual factors might explain the high prevalence rate and the severity of alexithymia among Chinese adult prisoners. Firstly, prison is a stressful place in which prisoners are foreboding from the outside and lacking opportunity to communicate with others [39, 40]. For example, the inmate has only two hours each day to socialize with other inmates and only two hours each month for family visits, during which the inmate and family members are separated by safety glass [72]. Therefore, the lack of communication and self-expressions can become routine and prisoners may find it harder to identify, describe and work with one’s own feelings and others. Secondly, compared with people living in the community, prisoners experienced much more physical health problems and mental health problems which are considered to act as triggering risk factors for the development, maintenance or exacerbation of alexithymia [42, 43]. Thus, prisoners in China have to face stress from both inside and outside, leading to a high incidence of severe alexithymia.

In this research, among the adult prisoners, as high as 96.2% of prisoners suffered from at least one traumatic experience in their childhood, furthermore, the prisoners sample had significantly higher alexithymia scores than adult norm of 2005. The prevalence rate and level of severity of childhood trauma among adult prisoners is more higher and severer than ones among other normal groups such as college students [73, 74]. It provides evidences for the view that the vast majority of prisoners have been victims of abuse or neglect as children, and childhood trauma is associated with later criminal behavior.

Moreover, among the adult prisoners, the prevalence of depression, anxiety and hopelessness was 81.5%, 53.4% and 85.8% respectively, this results were consistent with the findings of academic studies that have repeatedly documented high rates of negative emotional symptoms in this population [75–77]. For example, in a current study, 252 inmates in a Nigerian prison was surveyed and found that 72.6% and 77.8% were found to be positive for depression and anxiety symptoms respectively [75]. Thus, more attention should be paid for this special but neglected group in the future studies.

Another important finding of the study is that some socio-demographic factors are significantly associated with an increased or decreased likelihood of alexithymia against adult prisoners.

Firstly, the high level of education for the subjects was identified as a protective factor of alexithymia, which is consistent with previous studies [78, 79]. This is justified as those subjects with lower levels of education (primary or none) have much more difficulty in distinguishing and appreciating the emotions of others compared to higher educated subjects; meanwhile, educated subjects have greater capacity of psychological mindedness and emotional intelligence and able to decrease problems identifying, processing, describing, and working with one’s own feelings, which could contribute to protect individuals from alexithymia.

Secondly, adult prisoners tended to be with more symptoms of alexithymia when being in prison for over 120 months. However, this association was not significant after controlling for other factors. There are possible explanations for why prisoners with longer duration in prison didn’t show higher level of alexithymia. As the existing literature has indicated, there are two different perspectives on the nature of alexithymia. From one perspective, some researchers suggested that alexithymia is state-dependent and disappears after stressful situation has been evoked or experience has changed [61, 80, 81], From the other perspective, other researchers defined alexithymia as an enduring psychological trait that does not alter over time and remains persistent due to neurological defects or internalized object-relations systems which radically alter normal neuronal activity [3, 82, 83]. Based on the latter perspective, it seems that alexithymia is an enduring psychological trait showing a high degree of relative stability over time. Thus, individuals with longer duration of imprisonment didn’t show higher levels of alexithymia.

Thirdly, childhood trauma acted as a risk factor for alexithymia. Specifically, those subjects with trauma experience in childhood have a 2 to 4-fold risk of alexithymia compared to subjects without childhood trauma. This indicates that the more childhood trauma prisoners experienced, the more severe their symptom of alexithymia. This result is consistent with a growing literature showing that childhood trauma is a well-described risk factor for the development of several psychiatric disorders, including alexithymia [5, 56, 84–86]. Based on the results of recent research on the effects of maltreatment on brain development, repeated trauma in childhood may stunt growth of part of the brain involved in emotions, such as hippocampus. For example, Martin Teicher found that the volumes of three important areas of the hippocampus were reduced by up to 6.5% in people exposed to several instances of maltreatment, such as physical or verbal abuse from parents in their early years [87]. Therefore, it is possible for maltreated children to have some problems involving recognition, expression and understanding of emotions, which are the core characteristics of alexithymia.

Fourthly, negative emotional symptoms including depression, anxiety and hopelessness were three of the most consistent markers of alexithymia. This is consistent with previous studies indicating that a strong link exists between alexithymia and various negative emotional conditions [42, 88–90]. Both the primary alexithymia theory and the secondary alexithymia theory could explain or interpret it. Primary alexithymia theory considered alexithymia as a trait deficit in the capacity to process or regulate emotions using cognitive strategies. This deficit predisposes a person to develop a host of disorders related to poor affect regulation, including some of the anxiety and mood disorders; secondary alexithymia theory, however, considered alexithymia as a state response to distress including various negative emotions and mood disorders. In one sense, our conclusion is consistent with the view of primary alexithymia theory and the secondary alexithymia theory.

We should acknowledge some limitations in our study. First and foremost, our study was based on cross-sectional design, which is not possible to get a valid cause-and-effect relation between alexithymia and duration in prison, childhood trauma, and negative emotions. To clarify the causality, we need longitudinal data or panel data for further research. Secondly, although the clarity, comprehensiveness, and acceptability of questionnaires were verified among 32 selected prisoners, the present study is limited to self-report methodology, in which individual heterogeneity biases are not known. Fourth, some relevant potential factors that are important to alexithymia research were not included, such as participant’s adult trauma and psychiatric history. The investigation of these factors could be key directions for future research. In addition, the sample is mainly adult prisoners in our study and it is doubtful to extend the results to teenage prisoners or general population. Further research on teenage prisoners sample or general population can be conducted.

## Conclusion

The problems of psychological and mental health of adult prisoners are alarming these days. We made effort to explore the prevalence of alexithymia as well as its relationship with experience in prison, childhood traumas and negative emotions. The present study indicated that alexithymia was serious and widespread among adult prisoners in China. From the findings of this study, we concluded that alexithymia among adult prisoners can be explained by their level of education, experience of childhood traumas (emotional abuse and physical neglect) and symptoms of negative emotions (anxiety, depression and hopelessness). These findings have important policy or practical implications.

## Additional file

Additional file 1: Data used in this paper. Description of data: data of socio-demographic information and associated factors of alexithymia among adult prisoners in China. (XLS 1407 kb)

## Abbreviations

BAI: Beck Anxiety Inventory
BDI: Beck Depression Inventory
BHS: Beck Hopelessness Scale
CTQ: Childhood Trauma Questionnaire
DDF: Difficulties Describing Feelings
DIF: Difficulties Identifying Feelings
EOT: Externally Oriented Thinking
TAS-20: the 20-item Toronto Alexithymia Scale
